# Small GTPases control macropinocytosis of amyloid precursor protein and cleavage to amyloid-β

**DOI:** 10.1016/j.heliyon.2024.e31077

**Published:** 2024-05-11

**Authors:** Justin Chiu, Jordan M. Krupa, Claudia Seah, Stephen H. Pasternak

**Affiliations:** a Department of Physiology and Pharmacology, The Schulich School of Medicine and Dentistry, Western University, London, Ontario, Canada; b Neuroscience Program, The Schulich School of Medicine and Dentistry, Western University, London, Ontario, Canada; c Robarts Research Institute, The Schulich School of Medicine and Dentistry, Western University, London, Ontario, Canada; d Department of Clinical Neurological Sciences, The Schulich School of Medicine and Dentistry, Western University, London, Ontario, Canada

**Keywords:** Alzheimer disease, Amyloid precursor protein, RhoGTPases, Macropinocytosis, Lysosomes

## Abstract

The overproduction of the toxic peptide amyloid-beta (Aβ) generated from the cleavage of amyloid precursor protein (APP) is proposed to be a critical event in the development of Alzheimer's disease. Evidence suggests that the cleavage of APP occurs after its internalization from the cell surface. Previously, we identified a novel pathway for APP internalization, which trafficks cell surface APP directly to lysosomes by macropinocytosis, leading to its processing into Aβ. We also demonstrated that ADP-ribosylation factor 6 (Arf6) is required for the macropinocytosis of APP. Here, we characterized the roles of Arf6's downstream effectors Rac1, Cdc42 and RhoA. Both pharmacological inhibition and siRNA knockdown of these proteins reduced the amount of APP colocalized with LAMP1-labeled lysosomes without affecting APP transport to early endosomes. Decreases in the production of both Aβ40 and Aβ42 were also observed by ELISA in response to inhibitor treatment. These findings together demonstrate that Rac1, Cdc42 and RhoA are components of the mechanism regulating the macropinocytosis of APP and targeting these components can reduce the production of Aβ.

## 1 Introduction

Alzheimer's disease (AD) is characterized by the accumulation of plaques composed of the peptide amyloid-beta (Aβ) and neurofibrillary tangles (NFTs) composed of the protein tau [1]. Aβ is produced by the sequential enzymatic cleavage of the single pass transmembrane protein amyloid precursor protein (APP) [1]. To produce Aβ, APP is first cleaved in its extracellular domain by β-secretase (BACE) followed by cleavage of the protein within the transmembrane domain by an enzyme complex known as γ-secretase. This cleavage results in the release of the Aβ peptide, ranging in size from 38 to 43 amino acids.

Many studies have linked Aβ production with APP's internalization from the plasma membrane into the endosomal/lysosomal system. These studies demonstrated that cleavage of surface-labeled APP occurs following internalization [2] and inhibition of internalization by deletion of the cytoplasmic domain of APP and dominant negative mutants of dynamin decreased Aβ production and release [3,4]. Reducing the acidification of the endo-lysosomal system has also been shown to reduce Aβ production [[5], [6], [7]]. The rapid appearance of labeled Aβ in the media in cell surface labeling experiments led to the assumption that early endosomes were the main compartment for Aβ production [3].

A number of studies have also specifically implicated failure of lysosomes or the autophagic pathway in Aβ production [[8], [9], [10]]. Additionally, γ-secretase sub-units colocalize with APP within lysosomes and the acidic pH of lysosomes is optimal for β-secretase [11] and γ-secretase activity, as well as Aβ aggregation [12,13]. Prior work in our laboratory examined the process responsible for APP's internalization to lysosomes. We identified a novel pathway by which APP is trafficked directly to lysosomes, bypassing early and late endosomes [14] and is internalized into ruffled membranes and macropinocytic structures [15]. This process is enhanced by binding/crosslinking APP via antibody at the cell surface and accompanies internalization with the fluid phase marker dextran [14]. This pathway was disrupted by inhibition of actin polymerization with latrunculin B and by a dominant negative mutant of the small GTPase Arf6, which resulted in decreased Aβ production [14,15]. Together, these previous findings have suggested that macropinocytosis plays an important role in the transport of APP to lysosomes and Aβ production.

The purpose of the current study was to examine the roles of regulatory proteins downstream of Arf6 in macropinocytosis. Specifically, we examined the RhoGTPases Rac1, Cdc42, and RhoA [16]. These proteins have all been implicated in Aβ formation, but their mechanism of action with respect to cell surface APP trafficking was not known [[17], [18], [19]]. Here, we show that the mechanism by which they influence Aβ production is through macropinocytosis. To do this, we examined APP trafficking from the cell surface while inhibiting the activity of these proteins both pharmacologically and by siRNA knockdown. We observed a specific reduction in APP internalization to lysosomes but not early endosomes in both N2A cells and primary mouse cortical neurons. We also demonstrated that inhibition of these RhoGTPases also resulted in marked decreases in Aβ40 and Aβ42 production.

## 2 Materials and methods

### 2.1 Antibodies and reagents

Antibodies purchased were: mouse anti-HA (HA-7, H3663, 1:100, Sigma-Aldrich, MO, USA), mouse anti-Aβ (6E10, 803001, 1:100, Biolegend, CA, USA), rabbit anti-NCAM1 (EPR21827, ab2203609, 1:100, MA, USA), rabbit polyclonal anti-BACE1 (PA1-757, 1:200, Invitrogen, MA, USA), mouse anti-PSEN1 (APS-18, MA1-752, 1:100, Invitrogen, MA, USA), mouse anti-LAMP1 (1D4B, 1:400, deposited to the DSHB by August, J.T., DSHB, IA, USA), rabbit polyclonal anti-LAMP1 (L1418, 1:400, Sigma-Aldrich, MO, USA), rabbit polyclonal anti-Cdc42 (P8, sc-87, 1:1 000, Santa Cruz Biotechnology, TX, USA), rabbit polyclonal anti-Rac1 (C-14, sc-217, 1:1 000, Santa Cruz Biotechnology, TX, USA), rabbit polyclonal anti-RhoA (119, sc-179, 1:1 000, Santa Cruz Biotechnology, TX, USA), α-tubulin (B-5-2-1, T5169, 1:10 000, Sigma-Aldrich, MO, USA), HRP anti-mouse IgG secondary antibody (12–349, 1:10 000, Sigma-Aldrich, MO, USA) and HRP anti-rabbit IgG secondary antibody (#1706515, 1:5 000, Bio-Rad, CA, USA), anti-mouse IgG Alexa Fluro 488 (A11001, 1:1000, Invitrogen, MA, USA), anti-rabbit IgG Alexa Fluro 488 (A21206, 1:1000, Invitrogen, MA, USA), anti-mouse IgG Alexa Fluro 647 (A21235, 1:1000, Invitrogen, MA, USA), anti-rabbit IgG Alexa Fluro 647 (A31573, 1:1000, Invitrogen, MA, USA). SN56 cells were obtained from Dr. Jane Rylett (Western University). Neuro-2a (N2a) mouse neuroblastoma cells were purchased from ATCC (VA, USA). Zenon Alexa Fluor 647 Mouse IgG1 Labeling Kit were purchased from Life Technologies (CA, USA). EHT 1864, ML 141, SR 3677 inhibitors and dimethyl sulfoxide (DMSO) were purchased from Sigma-Aldrich (MO, USA). Human Aβ40 and Aβ42 ELISA assay kits were purchased from Life Technologies (CA, USA). Chloroquine diphosphate salt (C6628, Sigma-Aldrich, MO, USA) was dissolved in molecular-grade water. Dulbecco's modified Eagle's medium (DMEM), minimum essential media (MEM), fetal bovine serum (FBS), heat-inactivated fetal bovine serum, Hank's balanced salt solution (HBSS), penicillin, streptomycin, and trypsin-EDTA were all purchased from Gibco (CA, USA).

### 2.2 DNA constructs

The HA-tagged βAPP-CFP construct (HA-βAPP-CFP; Supplemental figure S2) used was described in our previous study and was demonstrated to traffick and behave in the same way as full-length APP [14]. LAMP1-YFP was a generous gift from Dr. Walter Mothes and re-cloned into mCherry. Rab5-mRFP was a generous gift from Dr. Stephen Ferguson. The untagged APP695 construct used was a generous gift from Dr. Jane Rylett. mApple-LAMP1-pHluorin-N-8 was a gift from Michael Davidson (Addgene plasmid # 54918). This construct contains a luminal pHluorin tag which is fluorescent at neutral pH and quenched at acidic pH, along with a cytoplasmic mApple tag. Together, this construct allows for fluorescent visualization of LAMP1 and lysosomal pH.

### 2.3 Cell culture and transfection

SN56 neuroblastoma cells were grown in DMEM, and Neuro-2a neuroblastoma cells were grown in MEM. Media was supplemented with 10 % (v/v) heat-inactivated FBS (Gibco), and 100 μg/mL penicillin/streptomycin (Gibco). Cells were kept in 25 cm^2^ or 75 cm^2^ flasks (Falcon) in an incubator at 37 °C in a humidified atmosphere containing 5 % CO_2_ and were split every 3–4 days. Cells were seeded at a density of 4 × 10^5^ cells/35-mm dish (MatTek, MA, USA) one day prior to transfection date. Cells were then transiently transfected with Lipofectamine 2000 or TurboFect according to manufacturer's instructions (Invitrogen, MA, USA). Following an incubation period of 24 h, N2a cells were differentiated by serum withdrawal; SN56 cells were also treated with 1 mM dibutyryl cyclic AMP (dbcAMP; Sigma, MO, USA) [20].

### 2.4 Neuronal culture

The culture of mouse cortical neurons was described in our previous study [15]. Pregnant CD1 mice were obtained from Charles River (MA, USA) under protocols approved by Western Ontario Animal Care Committee. Cortical neurons were prepared from mouse embryos and plated on poly-l-ornithine-coated plates. These cells were grown in Neurobasal medium supplemented with 1X B27 and 0.8X N2 supplements, 2 mM glutamax and 50 μg/mL penicillin/streptomycin (Invitrogen, MA, USA). Half of this media was replaced with fresh media every 3 days. Transfection typically occurred after five days in culture, utilizing lipofectamine 2000 as described above. These cells were also fixed and stained with NeuN to ensure these cultures contained 75 % neurons.

### 2.5 Inhibitor treatments

Following differentiation, cells received fresh serum-free medium at indicated concentrations of EHT 1864, ML 141, or SR 3677 dissolved in DMSO. 0.1 % DMSO (v/v) in serum-free medium was used as a vehicle control. Cells incubated with ML 141 were incubated for 1 h at 37 °C and 5 % CO2 before being subjected to internalization experiments. Cells incubated with SR 3677 were incubated for 6 h at 37 °C and 5 % CO2 before being subjected to internalization experiments. Finally, cells incubated with EHT 1864 were incubated for 18 h at 37 °C and 5 % CO2 before being subjected to internalization experiments. These incubation times were chosen based on prior studies utilizing these inhibitors [17,19,21].

### 2.6 Confocal microscopy

Imaging was performed on a Zeiss LSM-510 META (Jena, Germany) laser scanning microscope using a Zeiss 63 × 1.4 numerical aperture oil immersion lens. The optical section thickness was typically 1 μm. ECFP fluorescence was imaged using 458 nm excitation laser and a 458–480 filter set. Alexa Fluor 488 and EYFP fluorescence was visualized using a 488 nm excitation laser and a BP 500–550 filter set. mRFP and mCherry fluorescence were visualized using a 543 nm excitation laser and a LP 560 filter. Alexa Fluor 647 fluorescence was imaged using a 633 nm excitation laser, and a LP 650 filter.

### 2.7 Cell surface APP labeling

Anti-HA antibody (Sigma-Aldrich, MO, USA) or 6E10 (Biolegend, CA, USA) was labeled with Alexa Fluor 647 using a Zenon Alexa Fluor 647 Mouse IgG1 Labeling Kit (Life Technologies, CA, USA) according to manufacturer's instructions. For fixed-time course experiments, labeled antibody was incubated with cells in DMEM, for SN56 cells, or MEM, for N2a cells, on ice for 30 min. Cells were washed twice in HBSS pre-warmed to 37 °C. After washing, warm HBSS was added to the dishes and the cells were incubated at 37 °C and 5 % CO2 for indicated times and then fixed with 4 % paraformaldehyde on ice for 15 min. The cells imaged had normal morphology and good expression of both βAPP and compartment marker constructs. Experiments were replicated 3 or 4 times as indicated for each treatment/construct, with at least 10 or 15 cells sampled in each experiment and time point.

### 2.8 Aβ40 and Aβ42 ELISA

N2a cells were plated at a density of 5 × 10^5^ cells into each well of a 6-well plate with 2 mL of MEM, with the addition of 10 % FBS (v/v) overnight. The following day, cells were transfected with equal amounts of HA-βAPP-CFP for 24 h. For Aβ40 ELISAs following transfection, the cells were given 1 mL of fresh serum-free medium in each well and 10 μM of the indicated inhibitor or 0.1 % DMSO diluted into the medium and then incubated for 24 h. For Aβ42 ELISAs following transfection, the cells were given 1 mL of fresh serum-free medium in each well and 10 μM of the indicated inhibitor or 0.1 % DMSO diluted into the medium and then incubated for 48 h. After culture, 500 μL of medium was collected and assayed using an ultrasensitive human Aβ40 or Aβ42 ELISA kit (Life Technologies, CA, USA) according to the manufacturer's instructions. Experiments were replicated 3 times, and inhibitor-treated conditions were normalized against the control. Data was plotted and analyzed using GraphPad (MA, USA) Prism 6.0 software and an unpaired *t*-test with a 95 % confidence interval.

### 2.9 siRNA knockdown

SN56 or N2a cells were split as described in the cell culturing subsection. Stealth siRNAs (Invitrogen, MA, USA) were purchased for Rac1 (GCCUGCUCAUCAGUUACACGACCAA), and Cdc42 (CCUUUCUUGCUUGUUGGGACCCAAA). Silencer Select siRNA (Life Technologies, CA, USA) was purchased for RhoA (AGCCUUGAUAGUUUAGAAATT). Cells were transfected with Lipofectamine 2000 (Life Technologies, CA, USA) manufacturer's instructions. Cell lysates were collected 3 days after transfection and assayed by western blotting with a 1:1000 concentration of Anti-Rac1, Anti-RhoA, or Anti-Cdc42 antibodies.

For trafficking studies, cells were transfected with HA-βAPP-CFP, LAMP1-mCherryFP, and 10 nM of siRNA Negative Control, as well as 400 nM of Rac1 siRNA, 200 nM Cdc42 siRNA, or 50 nM RhoA siRNA depending on the experiment. Following 48 h of transfection, cells were differentiated for 1 day and then surface labeled with Alexa Fluor 488 Zenon-labeled anti-HA antibodies as previously described and allowed to internalize for 15 min at 37 °C. Cells were then fixed with 4 % paraformaldehyde. Following fixation cells were imaged using confocal microscopy.

### 2.10 Protein extraction and western blotting

SN56 or N2a cells were plated on 60 mm Falcon dishes (Corning, NY, USA) at a cell density of 1.5 × 10^6^ per 60 mm plate. The following day, plates were transfected with the appropriate DNA constructs and siRNA transcripts. Following 2 days of incubation and 1 day of differentiation, cells were washed in cold PBS and lysed with NP40 lysis buffer (20 mM Tris pH 8.0, 137 mM NaCl, 10 % glycerol, 1 % IGEPAL/NP40) for 30 min at 4 °C. Cells were then scraped, sonicated, and centrifuged at 14 000 rpm for 10 min at 4 °C to remove insoluble material. Protein quantification of supernatant was performed using a Pierce BCA protein assay kit (Life Technologies, CA, USA) according to manufacturer's instructions. Total cell lysates were separated in a 12 % SDS-PAGE gel by electrophoresis at 140 V for approximately 1 h and transferred onto polyvinylidene fluoride (PVDF) membranes using semi-dry transfer at 25 V for 30 min. Membranes were first probed with Rac1 (1:1000), Cdc42 (1:1000), RhoA (1:1000), or α-tubulin (1:10 000) antibodies, then incubated in HRP-mouse antibodies (1:10 000, Sigma, MO, USA), developed using ECL and imaged. Quantification images was done using Image Lab software.

### 2.11 Data quantification and analysis

Colocalization analysis was performed on confocal optical sections using Imaris 7.0.2 (Oxford Instruments, Abingdon, UK) with Imaris Colocalization module. Thresholds were set to select only the brightest 2 % of pixels in the HA-tagged βAPP channel as in Ref. [14], and the brightest pixels that demarcated a vesicle in the compartment marker channel (LAMP1 or Rab5), to ensure only lysosomes or endosomes were considered in analysis. Imaris then generated a colocalization percentage by determining the number of pixels (above threshold) of surface labeled APP that are colocalized with the lysosomal or endosomal marker. For experiments examining lysosomal pH using LAMP1-phLuorin-mApple, mean phLuorin signal intensity within mApple labeled lysosome was determined by creating 3D spots corresponding to mApple signal using Imaris. For BACE1 and PSEN1 colocalization with LAMP1, thresholds were set to demark puncta containing BACE or PSEN1 and LAMP1 as described above. 10–15 cells were quantified from multiple plates for each replicate. Experiments were repeated at least 3 times. Graphing and statistical analysis was performed using GraphPad Prism 6.0. Spot creation for determining endosome diameter was done using Imaris 10.0.1 (Oxford Instruments, Abingdon, UK) using their surpass spot creation tool.

## 3 Results

### 3.1 Inhibition of Rac1, Cdc42, or ROCKII reduces trafficking of APP to lysosomes in a dose-dependent manner

**Fig. 1:**
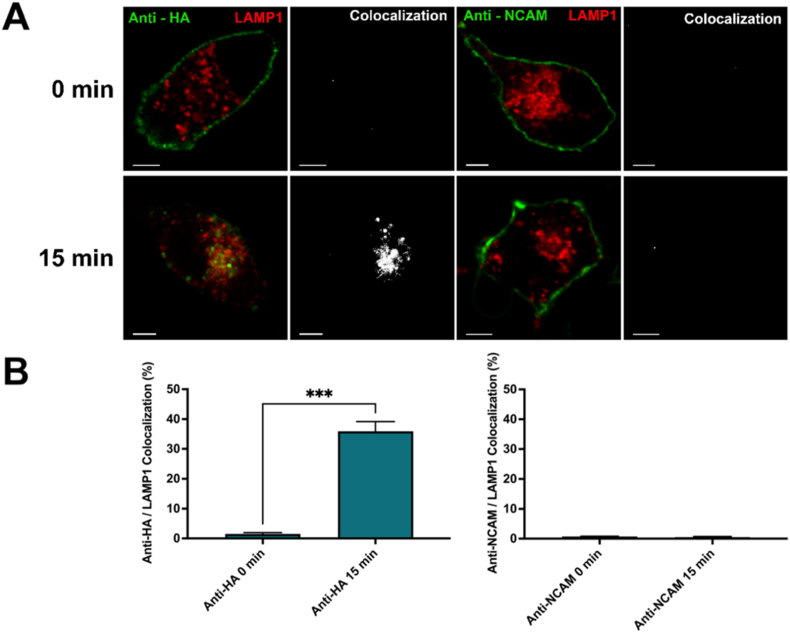
**Rapid internalization to lysosomes is specific to binding APP by antibody. A)** N2a cells were transfected with HA-βAPP-CFP, and LAMP1-mCherryFP (red). Cells were then incubated on ice with either anti-HA or anti-NCAM antibodies labeled with Zenon-Alexa Fluor 647 (green) for 30 min. Following this incubation, cells were either fixed immediately (0 min; top row) or placed in an incubator for 15 min at 37 °C (15 min; bottom row). Colocalization between HA-tagged APP and LAMP1 (white) can be observed after the 15-min incubation while there is little to no colocalization between NCAM and LAMP1 at any timepoint. **B)** Quantification of colocalization between anti-HA and LAMP1 (right) or anti-NCAM and LAMP1 (left) at 0 and 15 min. Percent colocalization was derived from at least 10 representative cells from 3 independent experiments (N = 3). Significant difference from 0 min is denoted by *** (p < 0.001) calculated by an unpaired two-tailed parametric *t*-test. *Scale bar* = 5 μm. (For interpretation of the references to colour in this figure legend, the reader is referred to the Web version of this article.)

First, the internalization of cell surface APP rapidly and directly to lysosomes was examined, as demonstrated in our previous studies [14,15]. N2a cells were co-transfected with the previously generated HA-βAPP-CFP construct (Fig. S2) [14], and LAMP1-mCherry was used to label lysosomes. Cells were then incubated with Zenon-Alexa Fluor 647 tagged anti-HA antibodies for 30 min on ice. These cells were either then immediately fixed (0 min) or fixed following a 15-min incubation at 37 °C (15 min; Fig. 1A). Fixation immediately following incubation on ice resulted in minimal colocalization between anti-HA and LAMP1-mCherry (1.53 % ± 0.7), followed by a significant increase in the amount of anti-HA colocalized with lysosomes (35.94 % ± 5.6; p < 0.001; Fig. 1B) after the 15-min incubation. To demonstrate that the internalization of APP was not due to a non-specific effect of incubating N2a cells with an antibody, the internalization of cell surface APP was compared to the cell surface protein neural cell adhesion molecule (NCAM). NCAM is abundant at the surface of neuronal cells and has previously been demonstrated to internalize by clathrin-mediated endocytosis (CME) [22]. N2a cells were transfected as described above, then incubated with anti-NCAM antibodies for 30 min while on ice, and then fixed at the same time points. No significant change in NCAM colocalization with LAMP1 was observed from 0 (0.726 % ± 0.14) to 15 min (0.52 % ± 0.34; Fig. 1B). Thus, these results suggest that the rapid internalization of HA-tagged APP to the lysosome by macropinocytosis is specific to APP rather than any potential non-specific cross-linking effects.

The small GTPases Rac1, Cdc42 and RhoA have all been demonstrated to regulate macropinocytosis in non-neuronal cell types [23]. To examine the role of these RhoGTPases in the macropinocytosis of APP in neuronal cells, their activity was blocked using pharmacological inhibitors. The Rac1 inhibitor EHT 1864 was chosen because previous studies show it can significantly decrease Aβ production *in vitro* [17]. Due to the lack of a selective RhoA inhibitor available, the ROCKII inhibitor SR 3677 was used to inhibit the downstream effects of RhoA activation. This inhibitor was also demonstrated to reduce Aβ production *in vivo* [19]. Finally, the Cdc42 inhibitor ML 141 was chosen for this study due to its high specificity [24].

N2a cells were co-transfected with HA-βAPP-CFP and LAMP1-mCherry and were treated with serum-free media (control), 0.1 % DMSO (vehicle control) and increasing concentrations of the three inhibitors (5, 10, 20 μM for EHT 1864 and ML 141; 5, 10, 25 μM for SR 3677). Cells were treated for 18 h for EHT 1864, 1 h for ML 141 and 6 h for SR 3677, based upon previously published experiments [17,19,21]. Cell-surface APP was labeled using anti-HA antibodies, then incubated for 15 min at 37 °C to permit internalization. This time point allowed for good visualization of APP in early endosomes and lysosomes but was brief enough so that surface-labeled APP was not trafficked to lysosomes by early endosomes [14]. The percentage of surface-labeled APP colocalized with LAMP1-mCherry-labeled lysosomes was then determined.

**Fig. 2:**
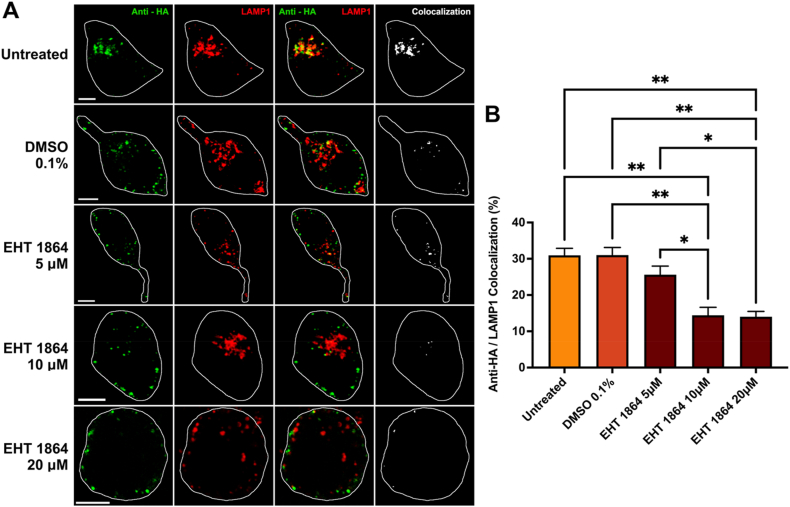
**Internalization of APP to lysosomes is decreased after Rac1 inhibition by EHT 1864**. **A)** Representative images for each experimental condition. N2a cells transfected with HA-βAPP-CFP and LAMP1-CherryFP (red). Cells were incubated with complete media (untreated), EHT 1864 or 0.1 % DMSO for 18 h and cell-surface APP was labeled with Zenon-Alexa Fluor 647 anti-HA antibodies (green) for 30 min on ice. Cells were then incubated for 15 min at 37 °C to allow for internalization. Following this, cells were fixed, imaged, and colocalization between anti-HA antibodies and LAMP1 was observed (white). **B)** Quantification of colocalization between anti-HA and LAMP1 channels for untreated control, 0.1 % DMSO, and EHT 1864 (5 μΜ, 10 μM, and 20 μM). Data are shown as mean percent colocalization ± SEM. Quantification was derived from at least 15 representative cells from each of 3 independent experiments (N = 3). Significant difference from control is denoted by * (p < 0.05), ** (p < 0.01) calculated by a one-way ANOVA and Tukey post-hoc test. *Scale bar* = 5 μm. (For interpretation of the references to colour in this figure legend, the reader is referred to the Web version of this article.)

**Fig. 3:**
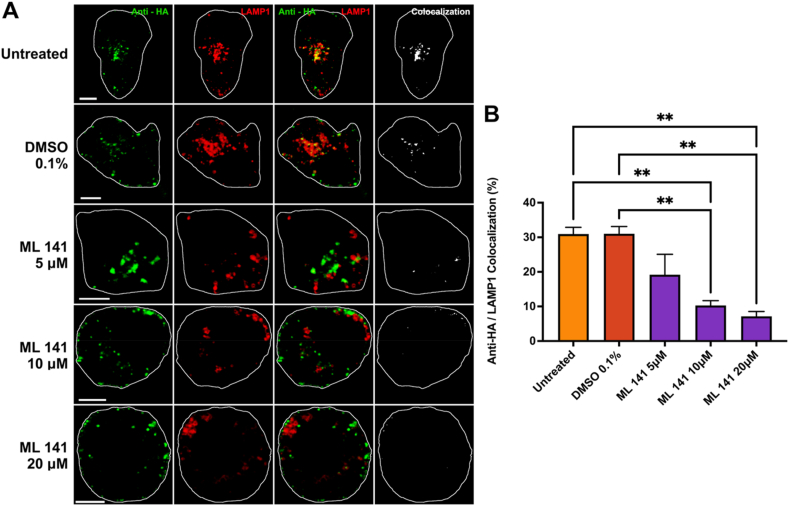
**Internalization of APP to lysosomes is decreased after Cdc42 inhibition by ML 141**. **A)** Representative images for each experimental condition. N2a cells transfected with HA-βAPP-CFP, and Lamp1-mCherryFP (red). Cells were then incubated with ML 141 or 0.1 % DMSO for 1 h, and immediately surface-labeled with Zenon-Alexa Fluor 647 anti-HA antibodies (green) for 30 min on ice, then incubated for 15 min at 37 °C. After 15 min of incubation at 37 °C, colocalization between anti-HA and LAMP1 was observed (white). **B)** Quantification of colocalization between anti-HA and LAMP1 channels for untreated control, 0.1 % DMSO, and ML 141 (5 μM, 10 μM, and 20 μM). Data are shown as mean percent colocalization ± SEM. Quantification was derived from at least 15 representative cells from each of 3 independent experiments (N = 3). Significant difference from control is denoted by * (p < 0.05), ** (p < 0.01) calculated by a one-way ANOVA and Tukey post-hoc test. *Scale bar* = 5 μm. (For interpretation of the references to colour in this figure legend, the reader is referred to the Web version of this article.)

**Fig. 4:**
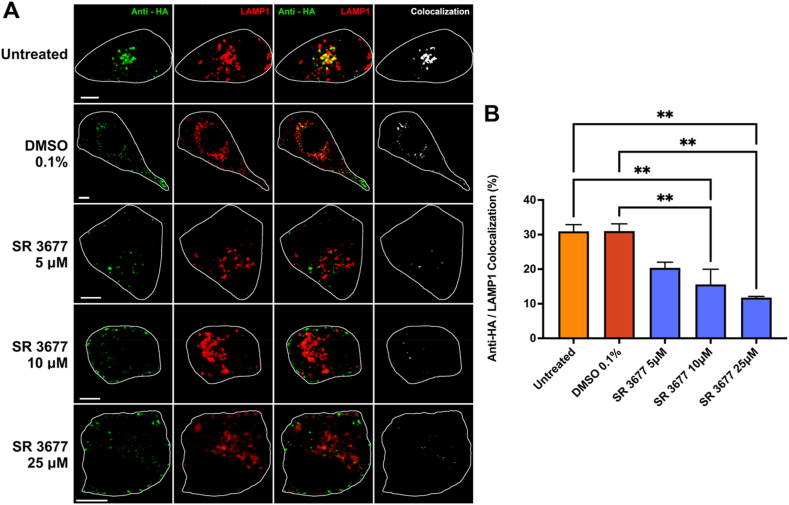
**Internalization of APP to lysosomes is decreased after ROCKII inhibition by SR 3677**. **A)** Representative images for each experimental condition. N2a cells transfected with HA-βAPP-CFP, and Lamp1-mCherryFP (red). Cells were incubated with SR 3677 or 0.1 % DMSO for 6 h, and immediately surface-labeled for APP with Zenon-Alexa Fluor 647 anti-HA antibodies (green) for 30 min on ice. Following this they were incubated at 37 °C for 15 min, and subsequently colocalization between anti-HA and LAMP1 was observed (white). **B)** Quantification of colocalization between anti-HA and LAMP1 channels for untreated control, 0.1 % DMSO, and SR 3677 (5 μM, 10 μM, and 25 μM). Data are shown as mean percent colocalization ± SEM. Quantification was derived from at least 15 representative cells from each of 3 independent experiments (N = 3). Significant difference from control is denoted by * (p < 0.05), ** (p < 0.01) calculated by a one-way ANOVA and Tukey post-hoc test. *Scale bar* = 5 μm. (For interpretation of the references to colour in this figure legend, the reader is referred to the Web version of this article.)

Inhibition of Rac1 using EHT 1864 caused a significant dose-dependent decrease in trafficking of APP to lysosomes after incubation (Fig. 2A). In these experiments, 31.0 % ± 1.9 of APP signal was colocalized with LAMP1 within 15 min in untreated cells (Fig. 2B). This was not significantly reduced by 0.1 % DMSO vehicle control (31.0 % ± 2.1) or 5 μM EHT 1864 (25.6 % ± 2.4) but was significantly reduced by 10 μM (14.4 % ± 2.2) and 20 μM (14.0 % ± 1.5) of EHT 1864 (p < 0.05; Fig. 2B). ML 141 mediated inhibition of Cdc42 also caused a significant dose dependent decrease in trafficking of APP to lysosomes following incubation (Fig. 3A). Cells which internalized APP without any treatment demonstrated 31.0 % ± 1.9 of APP colocalized with LAMP1 within 15 min (Fig. 3B). This was not significantly reduced by treatment with 0.1 % DMSO vehicle control (31.0 % ± 2.1) or 5 μM ML 141 (19.2 % ± 5.9) but was significantly reduced by 10 μM (10.3 % ± 1.4) and 20 μM (7.2 % ± 1.4) concentrations (p < 0.05; Fig. 3B). The ROCKII inhibitor SR 3677, also inhibited trafficking of APP to lysosomes in a dose dependant manner as well (Fig. 4A). In untreated cells, 31.0 % ± 1.9 of APP was colocalized with LAMP1 within 15 min (Fig. 4B). This was not significantly reduced by 0.1 % DMSO vehicle control (31.0 % ± 2.1) or 5 μM of SR 3677 (20.4 % ± 1.6) but was significantly reduced by 10 μM (15.6 % ± 4.4) and 25 μM (11.8 % ± 0.3) SR 3677 (p < 0.05; Fig. 4B). Thus, each of these inhibitors blocked direct cell-surface to lysosome transport of APP.

### 3.2 Inhibition of Rac1, Cdc42, or ROCKII does not affect trafficking of APP to early endosomes

**Fig. 5:**
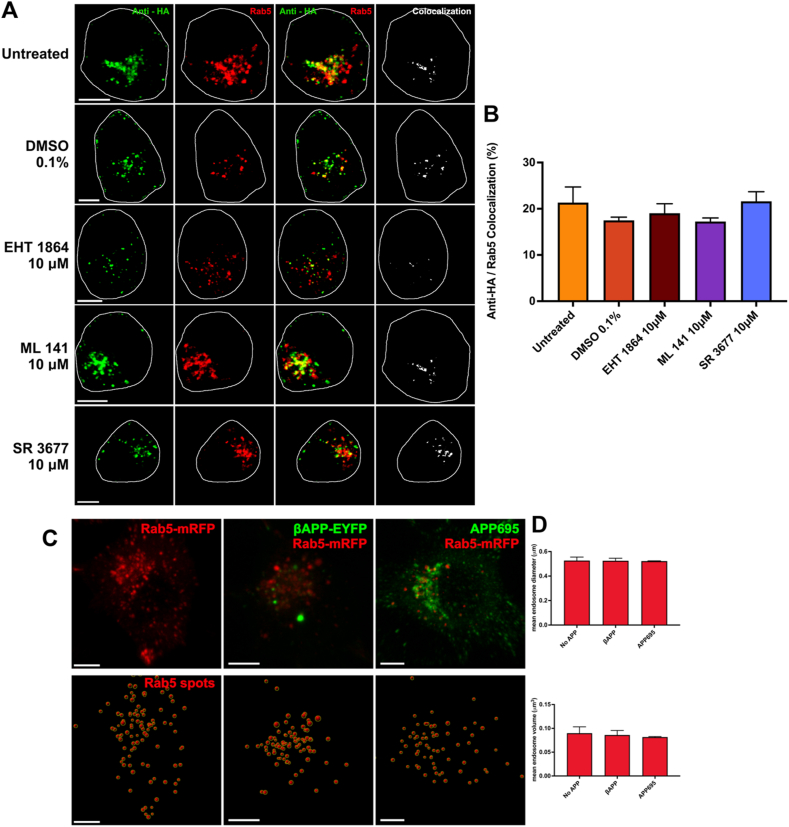
**Internalization of APP into early endosomes is unaffected after inhibition of Rac1, Cdc42, or ROCKII. A)** N2a cells transfected with HA-βAPP-CFP, and Rab5-mRFP (red). Cells were then incubated with 10 μM EHT1864, ML141, SR3677, 0.1 % DMSO, or serum-free media (control) for 18 h, and immediately surface-labeled with Zenon-Alexa Fluor 647 anti-HA antibodies (green) for 30 min. Cells were incubated for 15 min at 37 °C, colocalization can then be observed between anti-HA and Rab5 (white). **B)** Quantification of colocalization between anti-HA and Rab5 channels for untreated control, 0.1 % DMSO, and 10 μM EHT 1864, ML 141, and SR 3677. Data are shown as mean percent colocalization ± SEM. Quantification was derived from at least 15 representative cells from 3 independent experiments (N = 3). Significant difference from control is denoted by * (p < 0.05), ** (p < 0.01) calculated by a one-way ANOVA and Tukey post-hoc test. **C)** Maximum intensity projection images of N2a cells transfected with Rab5-mRFP (red) alone or with HA-βAPP-YFP or APP695 (green). Spots were created based on Rab5-mRFP signal using Imaris 10.0.1, which allowed endosome diameter and volume measurement based on Rab5 signal. **D)** Quantification of average mean spot diameter (μm) and volume (μm^3^) from 10 representative cells from 3 replicates (N = 3). No significant differences were found by a one-way ANOVA and Tukey post-hoc test. *Scale bar* = 5 μm. (For interpretation of the references to colour in this figure legend, the reader is referred to the Web version of this article.)

Because the internalization of APP to endosomes has long been implicated in Aβ production, the specificity of these inhibitors on direct lysosomal trafficking by macropinocytosis needed to be determined. To examine this, N2a cells were co-transfected with HA-βAPP-CFP and Rab5-mRFP to label early endosomes. Cells were left in serum free medium, treated with 0.1 % DMSO vehicle control or EHT 1864, ML 141 with SR 3677 for 18 h 10 μM was used for all inhibitors because this was the lowest concentration that achieved a significant reduction of direct lysosomal transport of APP. Cell surface APP was labeled as described above then allowed to internalize for 15 min, fixed and imaged. The co-localization of anti-HA and Rab5 channels was assessed to visualize cell surface APP internalized to early endosomes (Fig. 5A). Analysis showed that 21.3 % ± 3.4 of labeled APP was colocalized with Rab5-mRFP (untreated) and was not significantly different from treatment with 0.1 % DMSO vehicle control (17.5 % ± 0.7), EHT1864 (19.0 % ± 2.0), ML141 (17.3 % ± 0.8) and SR 3677 (21.6 % ± 2.1; Fig. 5B). These results suggest that inhibition of Rac1, Cdc42, or ROCKII does not affect APP trafficking through clathrin-mediated endocytosis, supporting that these inhibitors specifically inhibit direct lysosomal transport of APP by macropinocytosis.

It has been previously observed that the BACE cleavage product of APP, β-cleaved carboxy-terminal fragment of APP (βCTF), can accumulate and result in enlarged endosomes [25,26]. Given the truncated βAPP construct used in this study is only slightly larger than the βCTF, an experiment was performed to examine any effects of this construct on endosome size. Here, N2a cells were transfected with Rab5-mRFP construct alone, with βAPP, or with APP695. Cells were fixed and then imaged to assess the size of endosomes in cells overexpressing βAPP or APP695 compared to non-overexpressing cells (Fig. 5C). We found no significant difference between cells transfected only with the Rab5 marker compared to cells expressing either βAPP or APP695 by either diameter or volume (Fig. 5D). Demonstrating this further confirms the normal trafficking of our βAPP construct as outlined previously [14], and supports the notion that APP macropinocytosis is not a by-product of endosomal swelling with βCTF.

### 3.3 siRNA knockdown of Rac1, Cdc42 or RhoA decreases trafficking of APP to lysosomes

To confirm that the effects seen here are due to inhibition of the target proteins and not off-target effects, we utilized siRNA-mediated knockdown of Rac1, Cdc42 or RhoA using siRNA. siRNAs against the GTPases were transfected for 48 h with a mock transfection, a negative control siRNA, or siRNA against Rac1 (200 nM, 300 nM, and 400 nM), Cdc42 (200 nM, 300 nM, 400 nM), or RhoA (50 nM, 75 nM, 100 nM). Transfected SN56 cells were examined for Rac1 levels by Western blotting. Rac1 expression was reduced by 37.16 % ± 14.46, 28.96 % ± 14.46 and 51.56 % ± 14.46, respectively, with the 400 nM dose being the most effective (Fig. S1A). Cdc42 and RhoA expression were tested in N2a cells. The relative expressions of Cdc42 were 1.01 ± 0.09 (untreated), 0.32 ± 0.19 (200 nM), 0.30 ± 0.25 (300 nM), and 0.24 ± 0.16 (400 nM) (Fig. S1B). The 200 nM and 400 nM concentrations significantly lowered the expression of Cdc42 as compared to untreated cells (p < 0.05). For RhoA, relative expressions were 1.01 ± 0.09 (untreated), 0.31 ± 0.10 (50 nM), 0.47 ± 0.13 (75 nM), and 0.47 ± 0.17 (100 nM) (Fig. S1C). The 50 nM, 75 nM, and 100 nM concentrations all significantly lowered the expression of RhoA as compared to untreated cells (p < 0.05).

**Fig. 6:**
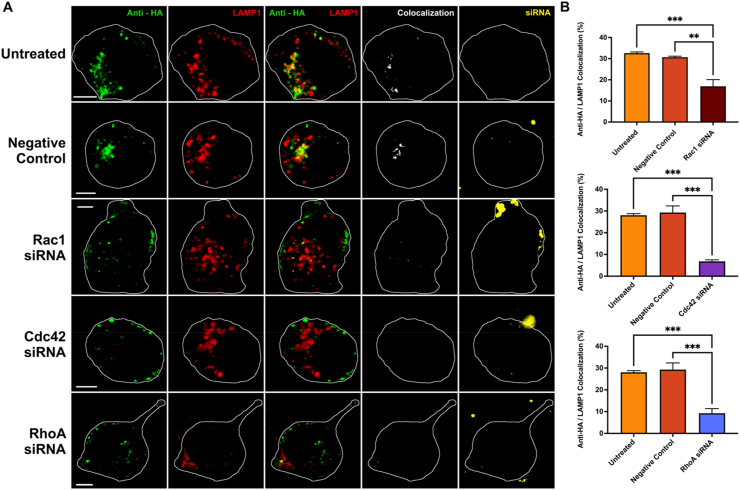
**Internalization of APP into lysosomes is decreased by siRNA knockdown of Rac1**, **Cdc42 or RhoA A)** Cells transfected with HA-βAPP-CFP, LAMP1-chFP (red), Stealth RNAi siRNA Negative Control Med GC-AlexaFluor 647 (yellow), along with Rac1 siRNA (MSS237709), Cdc42 siRNA (MSS247082), or RhoA siRNA (s119551). Cells were then differentiated and immediately surface-labeled with Zenon-Alexa Fluor 488 anti-HA antibodies (green) on ice, then incubated at 37 °C to allow internalization. After 15 min of incubation at 37 °C, colocalization between anti-HA and LAMP1 was observed (white). **B)** Quantification of colocalization between anti-HA and LAMP1 channels for mock transfection, negative control, and Rac1 siRNA (top), Cdc42 siRNA (middle), RhoA (bottom). Data are shown as mean percent colocalization ± SEM. Quantification was derived from at least 15 representative cells from 4 independent experiments (N = 4). Significant difference from mock is denoted by * (p < 0.05) calculated by a one-way ANOVA and Tukey post-hoc test. *Scale bar* = 5 μm. (For interpretation of the references to colour in this figure legend, the reader is referred to the Web version of this article.)

For internalization experiments, SN56 cells were co-transfected with HA-βAPP-CFP, LAMP1-mCherryFP, and Rac1 siRNA at 400 nM concentrations. A negative control siRNA conjugated to AlexaFluor 647 was also transfected into every plate at 10 nM except for the mock transfection plate to visualize transfected cells. The fluorescent control siRNA appeared in large bright puncta (Fig. 6A), likely a by-product of its concentration in the carrier used for transfection [27]. After transfection and differentiation, cell surface APP was labeled with anti-HA antibody on ice, then allowed to internalize. Co-localization between anti-HA and LAMP1 was assessed to determine the internalization of APP into lysosomes as above. For Rac1 siRNA experiments (Fig. 6A), untreated cells (32.6 % ± 0.5) and negative control siRNA (30.7 % ± 0.5) showed no significant differences in APP trafficking. However, the Rac1 siRNA treatment (16.9 % ± 3.2) showed a significantly lower (p < 0.05) amount of APP colocalized with lysosomes (Fig. 6B). For Cdc42 or RhoA siRNAs were used at 200 nM and 50 nM respectively. Colocalizations were 28.1 % ± 0.7 (untreated), 29.3 % ± 3.1 (negative control), 6.8 % ± 0.7 (Cdc42 siRNA) and 9.3 % ± 2.1 (RhoA siRNA) (Fig. 6B). In summary, siRNAs against Rac1, RhoA or Cdc42 each significantly reduced the transport of APP to lysosomes.

### 3.4 Inhibition of Rac1, Cdc42, or ROCKII reduces trafficking of APP to lysosomes in cultured cortical neurons

**Fig. 7:**
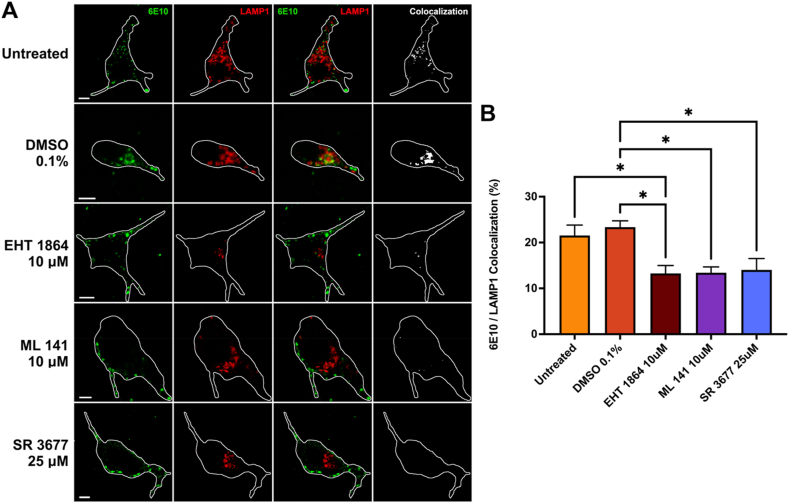
**Internalization of APP to lysosomes is reduced in mouse cortical neurons after treatment with GTPase inhibitors. A)** Representative images for each experimental condition. Mouse cortical neurons transfected with APP695, and LAMP1-mCherryFP (red). Neurons were incubated with EHT 1864, ML 141, SR 3677 or 0.1 % DMSO as vehicle control. Cell-surface APP was labeled with Zenon-Alexa Fluor 647 anti-Aβ antibodies (green) for 30 min on ice. Following this, neurons were incubated at 37 °C for 15 min, and subsequently colocalization between 6E10 and LAMP1 was measured (white). **B)** Quantification of colocalization between 6E10 and LAMP1 channels for control, 0.1 % DMSO, EHT 1864 10 μM, ML 141 10 μM, and SR 3677 25 μM. Data are shown as mean percent colocalization ± SEM. Quantification was derived from at least 15 representative cells for each from 3 independent experiments (N = 3). Significant difference from control is denoted by * (p < 0.05) calculated by a one-way ANOVA and Tukey post-hoc test. *Scale bar* = 5 μm. (For interpretation of the references to colour in this figure legend, the reader is referred to the Web version of this article.)

Next, the effect of inhibition on APP trafficking to lysosomes was examined in mouse primary cortical neurons. Cortical neurons were transfected LAMP1-mCherry and a construct transiently expressing the 695 amino acid human APP variant (APP695). APP695 is the APP variant preferentially expressed in neuronal cells [28]. These transfected neurons were treated with either 10 μM of EHT 1867, 10 μM ML 141, 25 μM of SR 3677 or 0.1 % DMSO as a vehicle control. Cell-surface APP was labeled with 6E10 antibodies on ice and incubated to allow internalization, as in the N2a experiments above. Co-localization of pixels from 6E10 and LAMP1 channels was used to assess APP internalization to lysosomes. Cortical neurons treated with inhibitors demonstrated a similar reduction in APP colocalization with LAMP1 (Fig. 7A) as demonstrated in N2a cells. In cells treated with DMSO, 23.4 % ± 1.4 of APP was colocalized with LAMP1. This was reduced by the inhibitors to 13.3 % ± 1.7 (EHT 1867), 13.4 % ± 1.3 (ML 141) and 14.0 % ± 2.5 (SR 3677) (p < 0.05; Fig. 7B). These results support that the RhoGTPases examined here regulate the internalization of APP to lysosomes via macropinocytosis in primary neurons.

### 3.5 Pharmacological inhibition of Rac1, Cdc42 or ROCKII significantly reduced secretion of Aβ40 and Aβ42

**Fig. 8:**
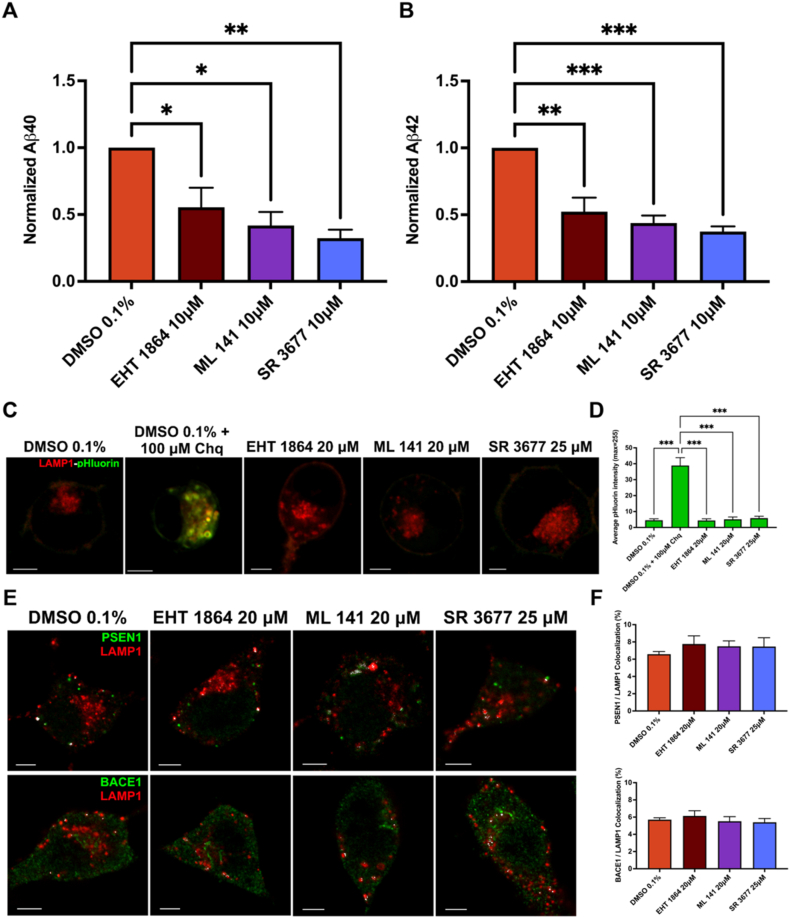
**Aβ40 and Aβ42 production is reduced after inhibition of Rac1, Cdc42, or ROCKII.** N2a cells transfected with HA-βAPP-CFP for 24 h then differentiated for 24 h. Differentiation medium was then replaced with fresh media containing 0.1 % DMSO, or 10 μM EHT1864 (Rac1 inhibitor), ML141 (Cdc42 inhibitor), or SR3677 (ROCKII inhibitor) for 24 h (Aβ40 ELISA) or 48 h (Aβ42 ELISA). Immediately afterwards, media samples were frozen and later analyzed using a human **A)** Aβ40 ELISA or **B)** Aβ42 ELISA according to manufacturer instructions. Data retrieved from 3 independent experiments done in duplicate (N = 3), significance is denoted by * (p < 0.05), ** (p < 0.01), *** (p < 0.001) as calculated by a one-tailed *t*-test. **C)** To control for any effects on lysosome pH by these inhibitors, N2a cells were transfected with a dual LAMP1-pHluorin-mApple reporter. Cells were subsequently treated with 20 μM EHT 1864 for 18 h, 20 μM ML 141 for 1 h, 25 μM SR 3677 for 6 h, 0.1 % DMSO or with 0.1 % DMSO +100 μM chloroquine (Chq) as a positive control to deacidify lysosomes. Treated cells were then imaged live to visualize the relative amounts of pH-sensitive pHluorin signal (green) and mApple signal (red). **D)** Quantification of pHluorin intensity. Mean pHluorin intensity within mApple labeled lysosomes was determined for at least 10 images per condition from 3 replicates (N = 3). A significant difference was found between 0.1 % DMSO +100 μM Chq and each condition denoted by *** (p < 0.001) as determined by one-way ANOVA and Tukey post-hoc test. **E)** To assess for any effects on PSEN1 or BACE localization, N2a cells were treated with 20 μM EHT 1864 for 18 h, 20 μM ML 141 for 1 h, 25 μM SR 3677 for 6 h or 0.1 % DMSO and stained with either PSEN1 or BACE1 (green) and LAMP1 (red). Colocalization between endogenous PSEN1 or BACE1 and LAMP1-labeled lysosomes was assessed (white). **F)** Comparison of quantified colocalization for either PSEN1 (top) or BACE1 (bottom) across conditions from at least 10 representative cells from 3 replicates (N = 3). No significant differences were found between treatments or DMSO control by one-way ANOVA and Tukey post-hoc test. *Scale bar* = 5 μm. (For interpretation of the references to colour in this figure legend, the reader is referred to the Web version of this article.)

After observing that siRNA knockdown and pharmacological inhibition of Rac1, Cdc42, or RhoA/ROCKII reduced lysosomal trafficking of APP, the effects of these inhibitors on Aβ production was examined. To measure this, human Aβ40 ELISA kits and human Aβ42 ELISA kits were used to analyze cell culture media following pharmacological inhibition. N2a cells were transfected with HA-βAPP-CFP and treated with 0.1 % DMSO vehicle control or each inhibitor at 10 μM. The amount of Aβ produced was normalized to the vehicle control. Production of Aβ40 was reduced by inhibitors to Rac1 (56.2 % ± 6.4), Cdc42 (49.3 % ± 17.7), or ROCKII (38.3 % ± 14.8) compared to control (p < 0.05; Fig. 8A). Similarly, the relative amount of Aβ42 was reduced by inhibitors to Rac1 (56.5 % ± 9.3), Cdc42 (48.6 % ± 8.0), or ROCKII (41.4 % ± 5.8) compared to control (p < 0.05; Fig. 8B). The Aβ42/Aβ40 ratio after inhibition of Rac1, Cdc42, or ROCKII remained unchanged compared to control.

Lysosomal pH and the localization of β-secretase (BACE1) and γ-secretase are known to influence the cleavage of APP to Aβ. The lysosome's acidic pH (pH 4.5) is optimal for the activity of both BACE and the catalytic subunit of γ-secretase presenilin-1(PSEN1). In addition, the localization of BACE1 has been demonstrated to affect both the production and clearance of Aβ [[11], [12], [13]]. To determine any effect of the RhoGTPase inhibitors on lysosomal pH, a dual LAMP1-pHluorin-mApple construct was used to label lysosomes (mApple signal) and assess their acidification (pHluorin signal is dark at neutral pHs). N2a cells were transfected with this construct and then treated with 20 μM EHT 1864 for 18 h, 20 μM ML 141 for 1 h, 25 μM SR 3677 for 6 h, 0.1 % DMSO or with 0.1 % DMSO +100 μM chloroquine (Chq; Fig. 8C). Chloroquine treatment was chosen as a positive control to mark pHluorin signal intensity as it neutralizes lysosomal pH [[5], [6], [7]]. The mean intensity of pHluorin within mApple labeled lysosomes was significantly higher (38.91 ± 8.5) than the DMSO vehicle control or inhibitor treatments (p < 0.001; Fig. 8D). Since the endocytic process in this study has been shown to bypass early and late endosomes to deliver APP directly to lysosomes [14], the effects of each GTPase inhibitor on the localization of PSEN1 and BACE1 to lysosomes was examined. N2a cells were treated as described above then endogenous PSEN1 or BACE1 was stained along with LAMP1 (Fig. 8E). Colocalization of either PSEN1 or BACE1 with LAMP1-labeled lysosomes was not significantly different between DMSO vehicle control and any of the inhibitor treatments (Fig. 8F). Together these results suggest that the decrease in Aβ production observed was specifically due to the inhibition of macropinocytosis of APP to lysosomes through Rac1, Cdc42 or ROCKII/RhoA inhibition.

## 4 Discussion

The internalization of cell surface APP has long been viewed as a key step in Aβ production, with many suggesting that APP cleavage events occur in early endosomes. Our laboratory has previously found that lysosomes are involved in the processing of APP [12], and that this process occurs in part through rapid internalization of APP from the cell surface [14]. This rapid internalization was identified as macropinocytosis and is mediated by the small GTPase Arf6 [15]. The purpose of the current study was to assess the contribution of the downstream of Arf6 effectors Rac1, Cdc42 and RhoA to the regulation of APP macropinocytosis and Aβ production. Here, we have demonstrated that both pharmacological inhibition and siRNA knockdown of Rac1, Cdc42, or ROCKII/RhoA can significantly reduce the amount of cell surface APP being transported to lysosomes without affecting internalization to early endosomes, and we show that these processes occur in primary cortical neurons. This reduced transport resulted in significantly reduced levels of Aβ40 and Aβ42, reinforcing the connection between lysosomal transport and APP processing.

Rac1, Cdc42 and RhoA have been previously demonstrated to be necessary for macropinocytosis and have been suggested to act downstream of Arf6 [16,23]. In the current study, we observed effects of inhibiting Rac1, Cdc42 and RhoA that were highly similar to those found previously with Arf6 mutations [15]. Of the three RhoGTPases examined in this study, Rac1 is the most strongly linked to macropinocytic function. Rac1 is essential for membrane ruffling and macropinosome formation, and requires both activation and deactivation for proper functioning [29]. There is a potential link with APP as well, as it has been shown that the adaptor protein Fe65, which binds to APP [30], stimulates both Arf6 and Rac1 activation [31]. Cdc42, like Rac1, has been heavily implicated in the formation of macropinosomes by mediating actin polymerization and reorganization. Cdc42 has been shown to bind neural-Wiskott-Aldrich syndrome protein (N-WASP), which binds and then activates the Arp 2/3 complex [32]. This results in the polymerization of actin filaments, forcing the plasma membrane into membrane ruffles and macropinosome formation [33]. Furthermore, Cdc42-mediated *N*-WASP function requires the binding of Abi1, an essential component of the WAVE protein complex that is activated by Rac [34]. RhoA is an essential regulator of neuronal morphology, often with effects opposing Rac1 [35,36]. However, RhoA activity has been demonstrated at membrane ruffles and macropinosomes [37,38]. While our data shows that knockdown or inhibition of RhoA has similar effects to Rac1 and Cdc42, it is possible that RhoA may be required for macropinosome closure rather than stimulating membrane ruffling. This is supported by a study which demonstrated that Arf6 activates Rac1 and RhoA in opposite manners to regulate spine formation in neurons [36]. Additionally, the RhoA effector ROCKII attenuates Rac1 activity at the leading edge of lamellipodia and membrane ruffles [39].

The exact mechanisms by which APP may mediate the recruitment of these RhoGTPases have yet to be elucidated. APP has long been thought to function as a cell surface receptor, and it has been demonstrated to have several binding partners and can interact with other cell surface proteins [28]. It seems likely then that APP could serve as a cell surface receptor and interact with extracellular ligands like a growth-factor receptor, form heterodimers with other membrane proteins, or homodimerize to signal the recruitment and activation of these RhoGTPases, as these mechanisms have all been linked to macropinocytosis [40]. It is important to note that much of the current study uses a truncated βAPP construct that lacks several ectodomain motifs that are likely to mediate these interactions. Considering that in this study, βAPP internalization was directly stimulated by binding and crosslinking it at the membrane using an antibody, it is likely that this results in the simulation of the effects of APP dimerization within the cytoplasmic domain. The cytoplasmic domain of APP contains a YENPTY motif that has long been considered an endocytosis signal [28]. One of the first binding partners demonstrated for this site was an adaptor protein known as Fe65 [28,41]. Fe65 has been demonstrated to act as a scaffold for the recruitment and activation of GTPases involved in actin dynamics [42]. Additionally, Fe65 has been implicated in Alzheimer's disease through its links to APP processing and mediating transcriptional activities [41]. Therefore, while the βAPP construct used in this study may lack the motifs responsible for the endogenous stimulation of APP macropinocytosis, its crosslinking by antibody may work by forcing its dimerization. This results in macropinocytosis similar to what we observed in this study with full-length APP695 in primary neurons and in previous studies [14,15]. Several heterodimer interactions with APP have been identified; one well-characterized example is sortillin-related receptor containing LDLR A repeats (sorLA) [43]. It has been demonstrated to regulate the trafficking of APP between the Golgi, endosomes and cell surface and, through this, modulate APP processing [44]. Given its lack of ectodomain, the use of a truncated APP construct likely affects its interaction with sorLA [44]. While this may have led to increased amounts of APP at the plasma membrane, given sorLA's low expression at the membrane [45], it seems unlikely to have played a role in the mechanism described in the study. It is worth noting that macropinocytosis APP internalization has so far only been observed in response to APP dimerization mediated by antibody crosslinking [14,15]. Thus, the role of heterodimers in APP macropinocytosis should be explored in the future. Investigating how interactions with APP drive the recruitment of macropinocytosis regulators and what aspects of APP structure and its respective binding partners are involved in stimulating endogenous APP macropinocytosis would further reveal the relationships between APP, RhoGTPases, macropinocytosis and Aβ production.

In this study, we observed that the pharmacological inhibition of the RhoGTPases Rac1, Cdc42, and RhoA (by ROCKII inhibition) significantly decreased both the amount of APP rapidly internalized to lysosomes and reduced the amount of both Aβ40 and Aβ42 present in the media. While the observed Aβ40/42 reductions seem to result from changes to APP macropinocytosis, the effects of the inhibitors on other aspects of Aβ production should be considered. In a previous study, the Rac1 inhibitor EHT 1864, had no downstream effects on BACE localization or activity and had only an indirect effect on the cleavage of C99 (BACE cleavage product) by γγ-secretase [17]. In this study, the authors suggested that this may be due to Rac1-specific effects on lipid rafts, where it has been suggested BACE and γγ-secretase cleavage can occur to produce Aβ [17]. This is particularly interesting as links between lipid rafts and macropinocytosis have been suggested, given observations that the recruitment and activation of Rac1 to produce membrane ruffling for macropinocytosis is cholesterol-dependent [[46], [47], [48]]. Combined with our results and others' observations that EHT 1864 inhibition of Rac1 prevents membrane ruffling [45], the observed reductions in Aβ production in response to Rac1 inhibition are likely due to changes to APP trafficking by macropinocytosis. RhoA and its downstream effectors have been demonstrated to affect APP processing outside of altering trafficking. ROCKI, another RhoA effector, was previously demonstrated to phosphorylate APP at S655, and its inhibition reduced Aβ production [49]. Additionally, the ROCKII inhibitor used in this study has been demonstrated to prevent phosphorylation of APP at T654, which affected APP processing by BACE, BACE localization and APP localization [19]. Contrary to our results, this study demonstrated increased BACE and APP localization to lysosomes following SR 3677 treatment [19]. This could be explained, in part, by the differing methods of examining APP. In our current study, cell surface APP is visualized and crosslinked using a fluorescently tagged antibody to follow the trafficking of this pool of APP specifically. In contrast, the previous study visualized the localization of whole cell APP following SR 3677 treatment. Future studies will have to elucidate the exact mechanism that mediates RhoA and downstream ROCK effects on Aββ production.

Given the literature demonstrating the accumulation of βCTF in enlarged or swollen endosomes, the size of endosomes was measured in response to the overexpression of each APP construct used in this study. No changes in endosome size were observed between non-overexpressing cells or cells overexpressing either the truncated or full-length APP constructs. This may be partly due to the transient nature of the overexpression of APP in the N2a cell line utilized in this study compared to studies utilizing either stably overexpressing N2a cells [25] or studies which utilized primary neurons, including human neurons derived from IPSCs [26]. Transient overexpression was chosen for the current study to avoid changing the cellular physiology of the cell lines used or influencing the trafficking of cell surface APP. Considering the known role of the endosomal protein Rab5 in macropinocytosis, the findings from these previous studies contain interesting connections with the findings of the current study and our previous studies demonstrating APP macropinocytosis [14,15]. Rab5 has been well established in non-neuronal cell types as a regulator of macropinocytosis and macropinosome formation [50,51]. Considering this, increased APP macropinocytosis could potentially explain the findings of accelerated endocytosis in response to stable APP overexpression and βCTF accumulation in Rab5 labeled compartments [25]. In fact, it has also been found that the overactivation of Rab5 in-vivo produces AD pathology in neurons, resulting in increased endocytosis in the absence of βCTF accumulation [52]. However, the enrichment of Rab5 in both early endosomes and macropinosomes makes understanding the relative contributions of macropinocytosis and early endosome dysfunction in explaining the accumulation of βCTF incredibly difficult. Developing new strategies to differentiate macropinosomes and early endosomes could lead to a deeper understanding of the mechanisms underlying the trafficking mechanisms which lead to βCTF accumulation and endosomal/lysosomal dysfunction in AD.

The RhoGTPases examined in this study have several interesting links to AD pathology. The first is the aforementioned studies on EHT 1864 and SR 3677, which demonstrated the reduction of Aβ production in response to Rac1 and ROCKII inhibition, respectively [17,19]. Within the AD brain, studies have demonstrated alterations in the amount and distribution of RhoA levels [53], and increased levels of Rac1 activity within the hippocampus of AD patients [54]. Additionally it has been shown Aβ increases the activity of Rac1 and Cdc42 and several of their downstream effectors in hippocampal neurons [18,55]. It is highly likely that the GEFs and GAPs which regulate the activity of these proteins play an essential role as well, as demonstrated by a recent study which showed that Cdc42GAP knockdown in mice recapitulated an AD phenotype, including observed increases in Aβ production [56]. Downstream effectors have also been closely linked to AD. The ROCK kinases downstream of RhoA are implicated in several AD pathophysiological mechanisms, such as APP and BACE trafficking, and are considered promising drug targets [57]. Downstream of Rac1, defects in the activity and localization of the p21-activated kinases (PAKs) have been observed in transgenic AD mouse models and resulted in cognitive impairments and dendritic spine alterations [58]. Localization of active PAK was increased at the plasma membrane and immunoprecipitated with its upstream effector Rac1 [59]. With the results of the current study in mind, the activity of the RhoGTPases and their downstream effectors play an important role in AD pathogenesis and warrant further study. Upstream proteins, such as Arf6 and Fe65, involved in macropinocytic regulation and activation of the RhoGTPases, have also been connected to AD pathophysiology. We previously demonstrated that the GTPase Arf6 regulates the macropinocytosis of APP and its subsequent processing to A β and that Arf6 is upregulated in regions of AD hippocampus [15]. Fe65, as well, has been observed to display altered transcript expression levels in the brains of AD patients [60]. Overall, each of the RhoGTPases examined in this study, and their associated upstream and downstream effectors, are demonstrably linked to AD pathophysiology and to macropinocytic/actin cytoskeleton regulation [40,42]. Our findings align with this current sentiment, and given the wide range of cellular processes that the RhoGTPases regulate, significant attention has been focused on targeting these proteins in treating neurodegenerative diseases [61]. Continued research should focus on investigating the specific cellular mechanisms mediated by the RhoGTPases in disease pathophysiology to better determine how their modulation could be utilized to develop new pharmacological therapies.

## 5 Conclusion

While the exact mechanism has been unknown, Rac1, Cdc42 and RhoA have all been previously implicated in regulating Aβ production. Here, we have demonstrated the underlying mechanism for this by demonstrating that the inhibition of these small GTPases blocks the direct trafficking of APP to lysosomes, but not to early endosomes, and reduced Aβ formation. Our results emphasize the importance of lysosomal trafficking in Aβ production and provide a mechanism of action for Rac1, Cdc42 and RhoA. Further studies of this pharmacologically targetable pathway could lead to therapies for Alzheimer's disease.

Funding sources: This work was funded by Canadian Institute for 10.13039/100005622Health Research (10.13039/501100000024CIHR) MOP 363457.

## Data availability

Data will be made available upon request.

S.P has received grant support from Zywie Bio LLC, Princeton, New Jersey, for work not related to this manuscript.

## CRediT authorship contribution statement

**Justin Chiu:** Investigation, Formal analysis, Data curation, Conceptualization. **Jordan M. Krupa:** Writing – review & editing, Writing – original draft, Visualization, Investigation, Formal analysis, Data curation. **Claudia Seah:** Methodology, Investigation, Data curation. **Stephen H. Pasternak:** Writing – review & editing, Supervision, Resources, Project administration, Methodology, Funding acquisition, Data curation, Conceptualization.

## Declaration of competing interest

The authors declare the following financial interests/personal relationships which may be considered as potential competing interests: Stephen Pasternak reports financial support was provided by The Canadian Institute for Health Research (10.13039/501100000024CIHR) Canada. Stephen Pasternak reports financial support was provided by Beaconbright Foundation. London, Canada. Stephen Pasternak reports a relationship with Zywie Bio LLC, Princeton NJ, USA that includes: shareholding, consulting or advisory and grant funding.
