# Supplementary material for: Small GTPases control macropinocytosis of amyloid precursor protein and cleavage to amyloid-β
Source: Heliyon. 2024 May 11;10(10):e31077. doi: 10.1016/j.heliyon.2024.e31077 (PMC11126852; doi:10.1016/j.heliyon.2024.e31077)
Supplement: Multimedia component 1 [file mmc1.docx]

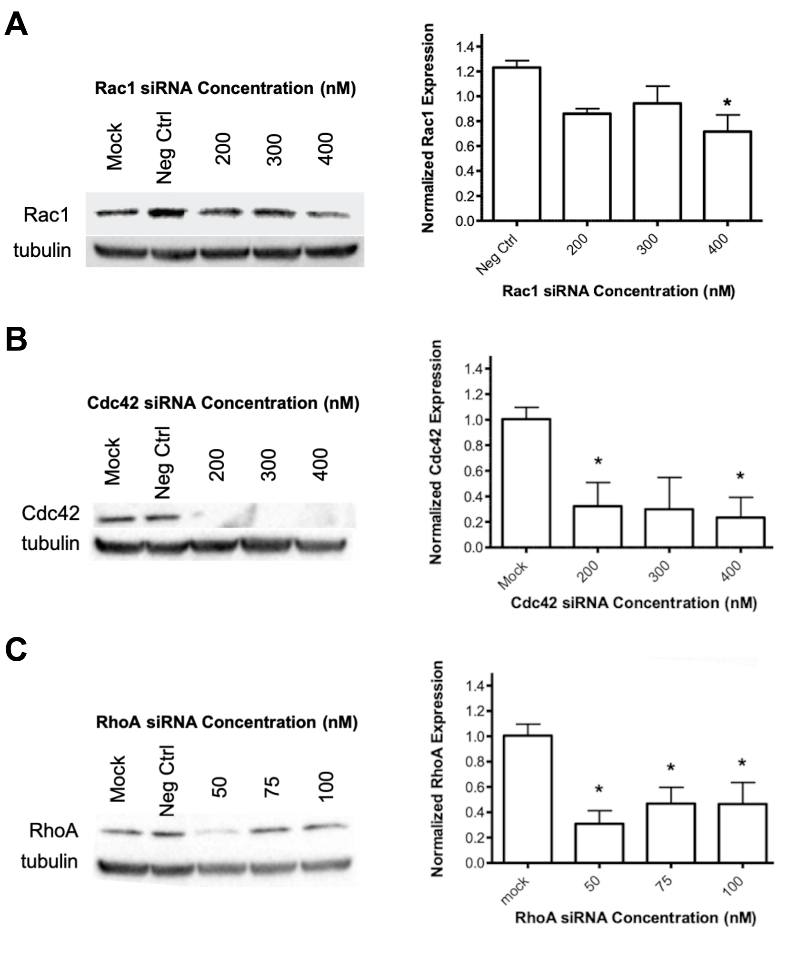


**Supplemental Figure S1.** siRNA mediated knockdown of Rac1, Cdc42 or RhoA. **A)** Left, Western blot image of siRNA knockdown of Rac1 in SN56 cells. Right, quantification of expression with negative control, or 200 nM, 300 nM, or 400 nM concentrations of Rac1 siRNA. Data retrieved from 3 replicates (N = 3), significance is denoted by * (p < 0.05) calculated by a one-way ANOVA and Tukey post-hoc test. **B)** Left, Western blot image of siRNA knockdown of Cdc42 in N2a cells. Right, quantification of expression with mock transfection, 200 nM, 300 nM, or 400 nM concentrations of Cdc42 siRNA. Data retrieved from 3 replicates (N = 3), significance is denoted by * (p < 0.05) calculated by a one-way ANOVA and Tukey post-hoc test. **C)** Left, Western blot image of siRNA knockdown of RhoA in N2a cells. Right, quantification of expression with mock transfection, 200 nM, 300 nM, or 400 nM concentrations of Cdc42 siRNA. Data retrieved from 3 replicates (N = 3), significance is denoted by * (p < 0.05) calculated by a one-way ANOVA and Tukey post-hoc test.


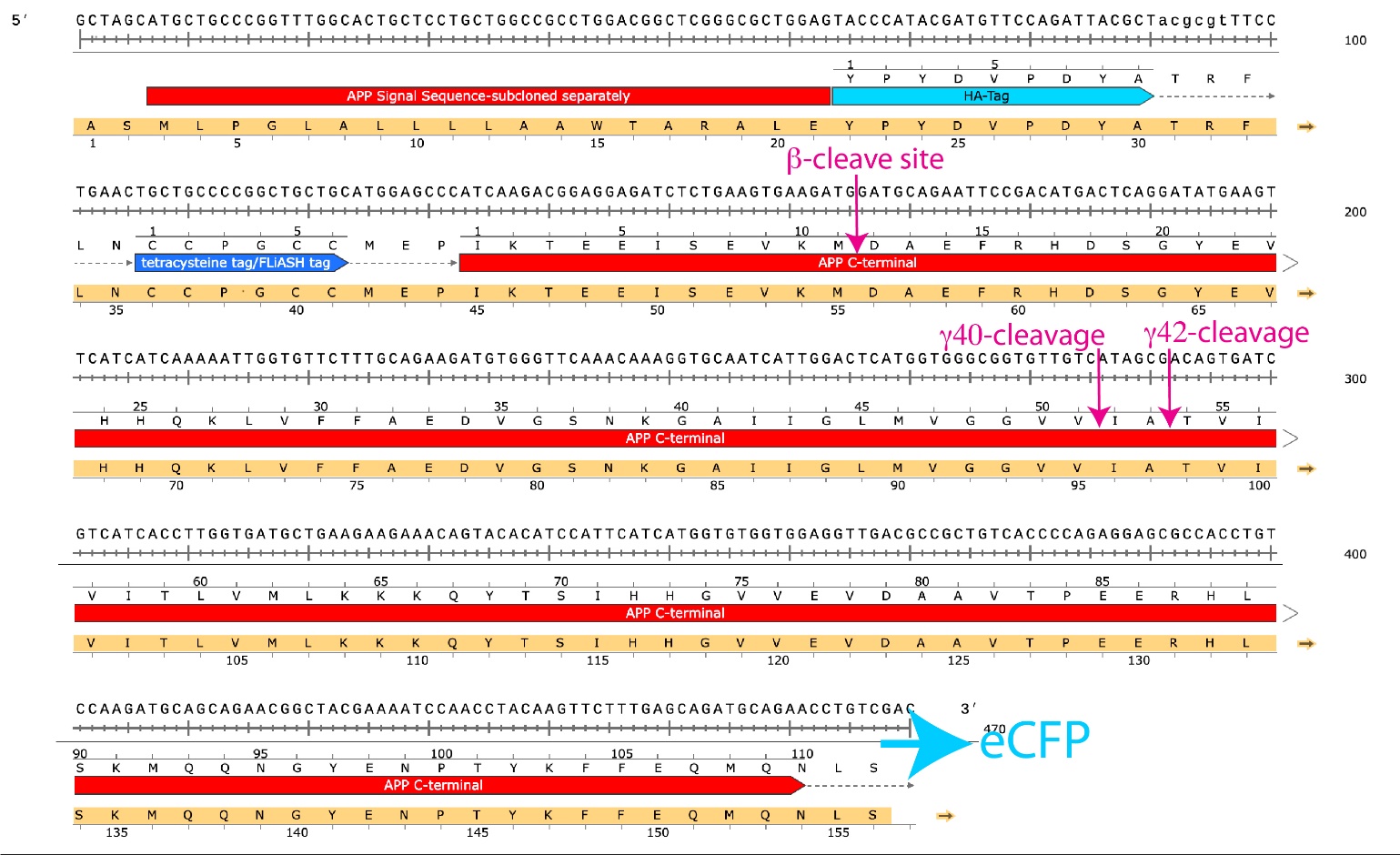


**Supplemental Figure S2.** Construct map of HA-βAPP-CFP construct including the C-terminal 121 amino acids of APP with an N-terminal HA-tag and C-terminal Cyan Fluorescent Protein (CFP) tag, referred to as (HA-βAPP-CFP) which was previously generated for APP internalization studies [14].*Figure was created using SnapGene (Dotmatics, MA, USA; www.snapgene.com)*
